# Susceptibility of Wild Canids to SARS-CoV-2

**DOI:** 10.3201/eid2809.220223

**Published:** 2022-09

**Authors:** Stephanie M. Porter, Airn E. Hartwig, Helle Bielefeldt-Ohmann, Angela M. Bosco-Lauth, J. Jeffrey Root

**Affiliations:** US Department of Agriculture, Fort Collins, Colorado, USA (S.M. Porter, J.J. Root);; Colorado State University, Fort Collins (A.E. Hartwig, A.M. Bosco-Lauth);; University of Queensland, St Lucia, Queensland, Australia (H. Bielefeldt-Ohmann)

**Keywords:** COVID-19, canid, *Canis latrans*, coronavirus disease, coyote, fox, red fox, SARS-CoV-2, *Vulpes vulpes*, wildlife, severe acute respiratory syndrome coronavirus 2, viruses, zoonoses, respiratory infections, United States, Australia

## Abstract

We assessed 2 wild canid species, red foxes (*Vulpes vulpes*) and coyotes (*Canis latrans*), for susceptibility to SARS-CoV-2. After experimental inoculation, red foxes became infected and shed infectious virus. Conversely, experimentally challenged coyotes did not become infected; therefore, coyotes are unlikely to be competent hosts for SARS-CoV-2.

Throughout the COVID-19 pandemic, multiple instances of natural infections with SARS-CoV-2 have been reported in pet dogs, likely after exposure to an infected human (*1*–*3*). Domestic dogs appear to be minimally susceptible to SARS-CoV-2, as indicated by experimental inoculations resulting in reverse transcription PCR–positive samples and low titer antibody responses but no clinical disease nor shedding of infectious virus (*4*,*5*).

The ability of SARS-CoV-2 to infect domestic dogs, in addition to several other species of carnivores, suggests that additional members of the canid family might be susceptible to infection. Wild canids, such as red foxes (*Vulpes vulpes*) and coyotes (*Canis latrans*), are of particular interest given how widely distributed these animals are, their frequent proximity to humans, and that they prey, scavenge upon, or otherwise interact with species demonstrated to be susceptible to SARS-CoV-2, including felids, skunks, rodents, and white-tailed deer (*6*,*7*). Foxes (species not specified) have been included in modeling efforts and serosurveillance studies aiming to predict animal hosts of SARS-CoV-2, but their ability to serve as hosts for SARS-CoV-2 remains unclear. 

Structural analysis of the ACE2 receptor in various animal species predicted that red fox ACE2 have the ability to bind to SARS-CoV-2; different models have predicted low-, medium-, or high-affinity binding (*8*–*10*). Two surveillance studies have sampled wild foxes and failed to detect antibodies against SARS-CoV-2; however, both studies were conducted early in the COVID-19 pandemic and did not exclude the possibility that foxes might become infected with and develop an immune response to SARS-CoV-2, only that spillover had not occurred in the examined animals in those locations at that time (*11*,*12*).

Because numerous carnivore species have proven to be susceptible to SARS-CoV-2, ascertaining the susceptibility of other wild carnivores to the virus, especially those species that are closely associated with humans, is a crucial step in understanding the role that wildlife might play in maintaining and transmitting SARS-CoV-2. The objective of this study was to assess 2 species of wild canids—red foxes and coyotes—for susceptibility to infection with SARS-CoV-2.

## The Study

We evaluated captive-reared, juvenile (3–5-month-old), mixed sex red foxes (3 female, 3 male) and coyotes (3 female, 1 male) for susceptibility to SARS-CoV-2. After approval by Colorado State University and National Wildlife Research Center Institutional Animal Care and Use Committees, we individually housed animals in an Animal Biosafety Level 3 (ABSL-3) facility at Colorado State University. Before inoculation, all animals were seronegative against SARS-CoV-2.

We diluted SARS-CoV-2 strain WA1/2020WY96 (obtained from BEI Resources, https://www.beiresources.org) in phosphate-buffered saline and instilled the solution into the nares of each animal. We immediately performed virus back-titration on Vero cells to confirm each animal received 5.1–6.0 log_10_ plaque-forming units of SARS-CoV-2.

We assessed all animals daily for attitude and signs of clinical disease, including lethargy, anorexia, ocular discharge, nasal discharge, sneezing, coughing, and dyspnea. We did not observe weight loss or elevated temperatures in any animals during the study. On 4 days postinfection (dpi), 1 red fox was observed to be lethargic, and all 3 red foxes remaining at 6 dpi were lethargic and sneezing. No other behavioral changes or clinical signs of disease were seen in any of the animals at any other time point.

**Figure 1:**
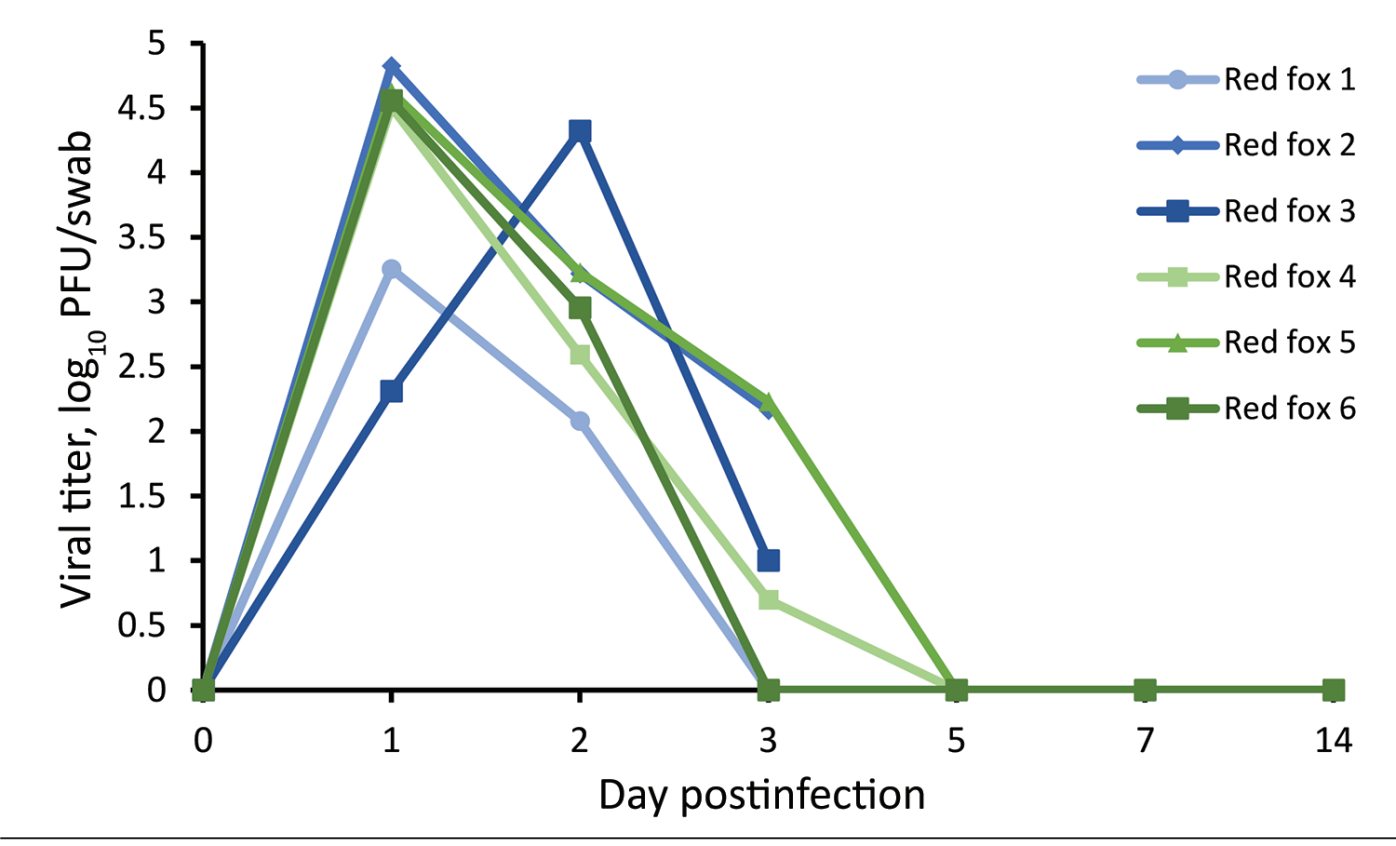
Oropharyngeal shedding of SARS-CoV-2 by red foxes (*Vulpes vulpes*) experimentally infected with SARS-CoV-2 as detected by plaque assay. Red foxes 1, 2, and 3 were euthanized at 3 days postinfection. PFU, plaque-forming unit.

**Figure 2:**
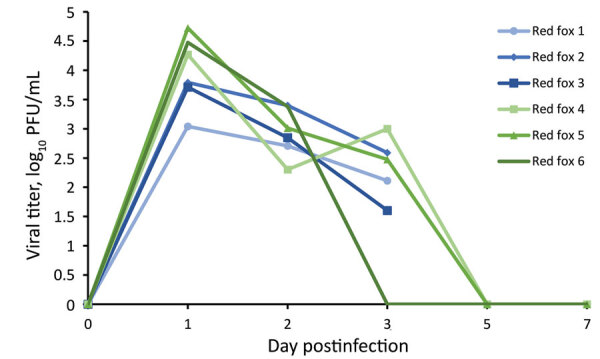
Nasal shedding of SARS-CoV-2 by red foxes (*Vulpes vulpes*) experimentally infected with SARS-CoV-2 as detected by plaque assay. Red foxes 1, 2, and 3 were euthanized at 3 days postinfection. PFU, plaque-forming unit.

We collected oral swab and nasal flush samples from each animal on 1, 2, 3, and 5 dpi and obtained additional oral swab samples on 7 and 14 dpi. Plaque assay revealed that all 6 of the red foxes shed infectious virus both orally (Figure 1) and nasally (Figure 2) starting at 1 dpi. Most of the red foxes were still shedding virus at 3 dpi (4/6 oral, 5/6 nasal); all shedding resolved by 5 dpi. We did not isolate infectious virus from any of the oral swabs or nasal flushes collected from any of the coyotes. Reverse transcription PCR confirmed the SARS-CoV-2 shedding profile of the red foxes and revealed that viral RNA was detected beyond the period that infectious virus was detectable (Table 1). The 2 dpi oral swab samples from all 4 coyotes were positive for SARS-CoV-2 RNA, albeit with high cycle threshold values (range 32–35) (Table 1). All other coyote oral swab samples were negative for SARS-CoV-2.

**Table 1:** Cycle threshold values by RT-PCR of oral swab samples from red foxes (*Vulpes vulpes*) and coyotes (*Canis latrans*) experimentally infected with SARS-CoV-2*

Animal	Cycle threshold value					
	1 dpi	2 dpi	3 dpi	5 dpi	7 dpi	14 dpi
Red fox 1	21.2	33.2	31.4			
Red fox 2	18.2	21.4	28.9			
Red fox 3	22.3	16.7	33.7			
Red fox 4	18.4	21.7	28.2	34.8	Undetected	Undetected
Red fox 5	17.9	18.3	28.4	Undetected	25.7	Undetected
Red fox 6	16.9	20.8	31.3	24.3	28.3	Undetected
Coyote 1	Undetected	35.0	Undetected			
Coyote 2	Undetected	32.6	Undetected			
Coyote 3	Undetected	34.0	Undetected	Undetected	Undetected	Undetected
Coyote 4	Undetected	33.1	Undetected	Undetected	Undetected	Undetected

*Red foxes 1, 2, and 3 and coyotes 1 and 2 were euthanized at 3 dpi. dpi, days postinfection; RT-PCR, revere transcription PCR.

We euthanized and necropsied one half of the animals (3 red foxes, 2 coyotes) at 3 dpi to evaluate tissues for acute viral burden and pathological changes. Infectious virus was isolated from the nasal turbinates of 2 of 3 red fox but not from any other tissues. None of the coyote tissues contained any infectious virus.

We collected blood for serologic testing weekly from the remaining animals until 28 dpi (red fox) or 30 dpi (coyote), at which point the animals were euthanized and necropsied. All of the red foxes held until 28 dpi showed a neutralizing antibody response beginning at 7 dpi; peak titers (1:80 or higher) were reached at 14 dpi (Table 2). None of the coyotes seroconverted.

**Table 2:** Antibody titers for red foxes (*Vulpes vulpes*) experimentally infected with SARS-CoV-2*

Animal	Antibody titer, PRNT_80_				
	Preinfection	7 dpi	14 dpi	21 dpi	28 dpi
Red fox 4	0	20	160	80	80
Red fox 5	0	20	80	80	80
Red fox 6	0	20	320	160	320

*dpi, days postinfection; PRNT**_80_**, 80% plaque reduction neutralization test.

On necropsy, we did not observe gross lesions in any animal. None of the fox tissues evaluated had histopathologic lesions attributable to SARS-CoV-2. We did not assess tissues from the coyotes histologically.

## Conclusions

The COVID-19 pandemic has been driven by human-to-human transmission of SARS-CoV-2, but animal species that are susceptible to infection with the virus represent a niche for viral maintenance and could potentially serve as a source for viral spillback into the human population, as has already been the case on mink farms (*13*). Peridomestic species are of particular interest because they presumably run the greatest risk for contracting the virus from humans.

We demonstrated that red foxes are susceptible to infection with SARS-CoV-2. All red foxes in this study shed infectious virus both orally and nasally for >3 days. Each of the red foxes held for 28 days displayed mild, self-resolving, clinical signs including lethargy and sneezing and developed neutralizing antibody responses beginning 7 dpi that persisted for the duration of the study. The antibody titers from red foxes were similar to what has been seen in experimentally infected domestic dogs (*4*). Conversely, coyotes appear not to be susceptible to infection with SARS-CoV-2; none of the animals in the study shed detectable virus nor seroconverted after challenge. Coyote oral swabs were positive for viral RNA on 2 dpi, but this result was not associated with isolation of infectious virus and likely represents either residual inoculum or an infection below the limit of detection. Hence, coyotes are unlikely to be competent hosts for SARS-CoV-2.

The animals thus far found to be susceptible to natural infection with SARS-CoV-2 have reflected results from experimental challenge studies, so it is reasonable to assume that our results can be extrapolated. Therefore, attention should be paid to red foxes when considering wildlife species that might serve as reservoir hosts for SARS-CoV-2. We demonstrated that SARS-CoV-2–infected red foxes shed infectious virus for multiple days in both oral and nasal secretions; consequently, the ability of red foxes to transmit SARS-CoV-2 to other susceptible animals should be investigated. Because red foxes commonly consume other species that are susceptible to infection with SARS-CoV-2, predator–prey interactions and scavenging might serve as avenues for interspecies transmission. Should wildlife species such as red foxes become established maintenance hosts of SARS-CoV-2, consequences could include effects on animal health, development of novel viral variants, and spillback into the human population.
